# Familial risk for depressive and anxiety disorders: associations with genetic, clinical, and psychosocial vulnerabilities

**DOI:** 10.1017/S0033291720002299

**Published:** 2022-03

**Authors:** Eleonore D. van Sprang, Dominique F. Maciejewski, Yuri Milaneschi, Bernet M. Elzinga, Aartjan T. F. Beekman, Catharina A. Hartman, Albert M. van Hemert, Brenda W. J. H. Penninx

**Affiliations:** 1 Amsterdam UMC, Vrije Universiteit, Psychiatry, Amsterdam Public Health Research Institute, Amsterdam, The Netherlands; 2 Department of Developmental Psychopathology, Behavioral Science Institute, Radboud University Nijmegen, Nijmegen, The Netherlands; 3 Institute of Clinical Psychology, Leiden University, Leiden, The Netherlands; 4 University of Groningen, University Medical Center Groningen, Interdisciplinary Center Psychopathology and Emotion regulation, Department of Psychiatry, Groningen, The Netherlands; 5 Department of Psychiatry, Leiden University Medical Center, Leiden, The Netherlands

**Keywords:** Anxiety disorder, clinical vulnerability, depressive disorder, family history, polygenic risk, psychosocial vulnerability

## Abstract

**Background:**

In research and clinical practice, familial risk for depression and anxiety is often constructed as a simple Yes/No dichotomous family history (FH) indicator. However, this measure may not fully capture the liability to these conditions. This study investigated whether a continuous familial loading score (FLS), incorporating family- and disorder-specific characteristics (e.g. family size, prevalence of depression/anxiety), (i) is associated with a polygenic risk score (PRS) for major depression and with clinical/psychosocial vulnerabilities and (ii) still captures variation in clinical/psychosocial vulnerabilities after information on FH has been taken into account.

**Methods:**

Data came from 1425 participants with lifetime depression and/or anxiety from the Netherlands Study of Depression and Anxiety. The Family Tree Inventory was used to determine FLS/FH indicators for depression and/or anxiety.

**Results:**

Persons with higher FLS had higher PRS for major depression, more severe depression and anxiety symptoms, higher disease burden, younger age of onset, and more neuroticism, rumination, and childhood trauma. Among these variables, FH was not associated with PRS, severity of symptoms, and neuroticism. After regression out the effect of FH from the FLS, the resulting residualized measure of FLS was still associated with severity of symptoms of depression and anxiety, rumination, and childhood trauma.

**Conclusions:**

Familial risk for depression and anxiety deserves clinical attention due to its associated genetic vulnerability and more unfavorable disease profile, and seems to be better captured by a continuous score that incorporates family- and disorder-specific characteristics than by a dichotomous FH measure.

## Introduction

Depressive and anxiety disorders are highly prevalent disorders with a substantial impact on public health (Vos et al., 2012). One of the strongest risk factors for depressive and anxiety disorders is a family history (FH) of these disorders, with a two-fold increased risk in patients' first-degree relatives as compared to healthy controls (Levinson, 2005; Micco et al., 2009; Rasic, Hajek, Alda, & Uher, 2014). Familial risk represents the integration of an underlying genetic vulnerability as well as enhanced risk due to familial clustering of unfavorable family circumstances in (early) life (Smoller, 2016). In light of the serious impact of depressive and anxiety disorders, there is a clinical need for identification of patients at risk of poorest outcome (Milne et al., 2009).

Familial risk for depression and anxiety is generally constructed as a simple dichotomization [hereafter referred to as family history (FH)] based on the presence (FH+) or absence (FH−) of a disorder in one or more relatives (e.g. see Milne et al., 2008). However, despite its status as an established risk factor for psychopathology, findings from previous studies in clinical samples investigating associations of FH with genetic, clinical, and psychosocial vulnerabilities for depression and anxiety have been inconsistent. For instance, studies have failed to find an association between FH and a genome-wide polygenic risk score (PRS) for major depression even though both are considered as indices of genetic vulnerability (Van Loo et al., 2018; Verduijn et al., 2017). Some studies showed that FH+ is associated with more severe and longer duration of illness and younger age of onset (e.g. Holma, Melartin, Holma, Paunio, & Isometsä, 2011; Husain et al. 2008; Seguí et al. 1999; Tozzi et al. 2008) while others found no association (Johnson, Andersson-Lundman, Åberg-Wistedt, & Mathé, 2000; Lamers et al. 2011a). Moreover, several personality traits (e.g. neuroticism, introversion, external locus of control; Docherty et al. 2017; Kotov, Gamez, Schmidt, & Watson, 2010) and depressive/anxiety cognitions (e.g. hopelessness, rumination, anxiety sensitivity; Aldao, Nolen-Hoeksema, & Schweizer, 2010; Dong, Liu, Oei, Cui, & Xiao, 2018; Gotlib, Joormann, & Foland-Ross, 2014; Maciejewski, Hillegers, & Penninx, 2018; Naragon-Gainey, 2010) have been suggested as endophenotypic traits underlying depression and anxiety. Yet, only neuroticism and social vulnerabilities, such as childhood trauma and negative life events, have been investigated in relation to FH, again with mixed results (neuroticism positively associated: Holma et al., 2011; not associated: Duggan, Sham, Minne, Lee, & Murray, 1998; social vulnerabilities positively associated: Jansen et al. 2016; Zimmermann et al., 2008, not associated: Manfro et al. 1996).

These inconsistent findings may be due to the use of a dichotomous indicator that, in a highly heterogeneous group of affected persons (Nandi, Beard, & Galea, 2009), may not fully capture the liability to depression and anxiety (Corfield, Yang, Martin, & Nyholt, 2017). For instance, independent of being categorized as having FH+ or FH−, affected persons may have had a single 2-week episode or chronic depression/anxiety with multiple episodes, stressing that heterogeneity. By default, a dichotomous indicator contains less information and therefore less statistical power to differentiate in terms of associated factors than a continuous indicator (Cohen, 1983). Specifically, it fails to take into account informative factors of familial risk for psychopathology (Milne et al., 2008), such as family size, number of affected family members, and their age. For instance, younger parental age of onset and having two (instead of one) affected parents/first-degree relatives have been shown to further increase the risk for psychopathology (Havinga et al., 2017; Wilde et al., 2014). A continuous indicator of familial risk that takes these aspects into account may therefore better reveal a person's vulnerability for psychopathology (Derks, Verweij, Kahn, & Cahn, 2009).

Few studies investigating clinical samples used a continuous indicator of familial risk for psychopathology. One study by Klein, Shankman, and Rose (2008) found that greater familial loading for depression predicted more severe depression symptoms during 10-year follow-up. Although the used continuous indicators of familial risk incorporated several family-specific characteristics (number, gender, and availability of direct interviews), no disorder-specific characteristics were taken into account. In contrast, three other studies used an algorithm created by Verdoux et al. (1996) to generate a continuous familial loading score (FLS) for psychopathology that incorporated both family- and disorder-specific characteristics: family size, number of affected family members, age of the relatives, a disorder's age range in which most first onsets appear (age of onset), and a disorder's lifetime prevalence (both for persons with FH+ and persons with FH−). The FLS showed predictive validity for several clinical outcomes (e.g. more severe symptoms or earlier age of onset) in psychotic patients (Verdoux et al., 1996) and children of bipolar parents (Hillegers et al., 2004; Wals et al., 2004). However, none of these studies directly compared their results to that of a dichotomous indicator.

Taken together, in clinical samples (i) familial risk for depression and/or anxiety is often constructed as a dichotomous indicator, rather than as a more comprehensive continuous indicator that incorporates both family- and disorder-specific characteristics known to be informative of familial risk (Milne et al., 2008), (ii) the evidence for associated genetic, clinical, and psychosocial vulnerabilities is mixed, and (iii) no previous studies directly compared the performance of a continuous indicator to that of a dichotomous indicator in terms of such associated vulnerabilities. This is however important, because many researchers and clinicians rely on measures of familial risk to potentially identify those patients at risk of poorest outcome. Therefore, this study aimed to investigate whether a continuous FLS, constructed according to the algorithm by Verdoux et al. (1996) which takes into account a wide range of family- and disorder-specific characteristics, is associated with genetic, clinical, and psychosocial vulnerabilities in persons that are lifetime affected with depressive and/or anxiety disorders. Second, we examined whether the continuous FLS provides a more comprehensive indicator of familial risk than the dichotomous FH measure by testing whether the FLS is associated with these vulnerabilities over and above FH. Considering the high comorbidity (Lamers et al., 2011b) and shared etiology of depression and anxiety (Mathew, Pettit, Lewinsohn, Seeley, & Roberts, 2011), FLS/FH indicators were determined for depression and/or anxiety combined.

## Methods

### Sample

Data were derived from the Netherlands Study of Depression and Anxiety (NESDA), which is an ongoing longitudinal cohort study of 2981 adults (2319 participants with a lifetime diagnosis of depressive and/or anxiety disorders and 652 healthy controls) aged 18–65 years. Between 2004 and 2007, participants were recruited from various settings [i.e. primary care practices (54.0%), specialized mental health institutions (27.1%), and the general population (18.9%)]. Participants were assessed at baseline, and 1-, 2-, 4-, 6-, and 9-year follow-up. All participants provided written informed consent. A detailed description of the NESDA study design has been reported elsewhere (Penninx et al., 2008).

The present study is based on lifetime-affected persons participating in the 9-year follow-up of NESDA (with a response rate of 69.4%, *N* = 2069) as this assessment included detailed FH assessment with the Family Tree Inventory (FTI, see below; Fyer & Weissman, 1999). A focus on lifetime-affected persons reduced heterogeneity as disease status itself then does not play a confounding role, and it best reflects the population generally asked about FH in practice. Of the 2069 participants at the 9-year follow-up, 396 were excluded because of no lifetime diagnoses of depressive (i.e. MDD and dysthymia) and/or anxiety disorder (i.e. panic disorder with or without agoraphobia, generalized anxiety disorder, social phobia, and agoraphobia) using the DSM-IV-based Composite International Diagnostic Interview (CIDI, version 2.1; Wittchen, 1994). The presence of lifetime diagnoses was based on data collected from baseline, 2-, 4-, 6-, and 9-year CIDI interviews, and indicated the presence of current depression and/or anxiety or diagnoses earlier in life. A further 248 were excluded due to missing data on the FTI, leaving 1425 participants for the present analyses. Attrition was low: 1378 participants (96.7%) had data on all or all but one of the total of six assessed waves (exact attrition rates can be found in online Supplementary Appendix S1). At 9-year follow-up, lifetime-affected persons with missing FTI data were more often female [191/248 (77.0%) female] than lifetime-affected persons with valid FTI data [949/1425 (66.6%) female; χ^2^(1) = 10.56, *p* < 0.001], but did not significantly differ in age and years of education.

### Materials and measures

#### Dichotomous family history (FH) indicator

FH information was obtained at 9-year follow-up by interviewing participants on the occurrence of depression and/or anxiety in their first-degree relatives (i.e. biological parents and siblings) using an extended version of the FTI (exact questions can be found in online Supplementary Appendix S2; Fyer & Weissman, 1999). First, participants were asked broad, simple questions on whether they had ever recognized a depressive or anxiety episode in their first-degree relatives (one question for each condition). Previous studies showed that affected persons tend to overestimate the presence of the same psychiatric disorders in their relatives (e.g. see Milne et al. 2008; Vandeleur et al. 2008, 2015). This was also the case in NESDA: at baseline, when FH was assessed using a single Yes/No question, FH was likely substantially overestimated with 79.2% of persons with lifetime depression and/or anxiety (Lamers et al., 2011a) and 71.9% of healthy controls reporting FH+ (Kruijt et al., 2013). These numbers are unlikely high considering the Dutch population-based lifetime prevalence of the disorders of 26.8% (as assessed by the Netherlands Mental Health Survey and Incidence Study; De Graaf, Ten Have, Van Gool, & Van Dorsselaer, 2012) and may have diluted associations of FH with clinical outcomes as seen in previous work on NESDA (Lamers et al., 2011a; Penninx et al., 2011; Verduijn et al., 2017; Vreeburg et al., 2010). For these reasons, in the assessment used for the present study, possible familial depression and anxiety was then validated for each first-degree relative using follow-up questions (e.g. presence of core symptoms, restrictions due to complaints, treatment, and hospital/psych ward admissions). A first-degree relative was only considered to be affected if participants endorsed (i) at least one question on presence of depressive/anxiety episodes, core symptoms, or restrictions and (ii) at least one question on receiving treatment or being admitted into a hospital/psych ward for that disorder for that relative. This information was used to determine a dichotomous FH Yes/No indicator, with participants reporting one or more affected first-degree relatives considered to have FH+.

#### Continuous familial loading score (FLS)

A FLS for depression and/or anxiety was determined using an algorithm originally designed by Verdoux et al. (1996) for psychotic disorder, schizophrenia, and affective disorder, with higher scores indicating more familial loading for a disorder. This method calculates a continuous score of familial risk while taking into account family size, number of affected first-degree relatives, age of the first-degree relatives, a disorder's age range in which most first onsets appear, and a disorder's lifetime prevalence. Specifics on participants' relatives and family were obtained at 9-year follow-up via the FTI (Fyer & Weissman, 1999). A short description of the calculation of the FLS for depression and/or anxiety will be given, the more detailed procedure can be found in online Supplementary Appendix S3.

**Fig. 1.**
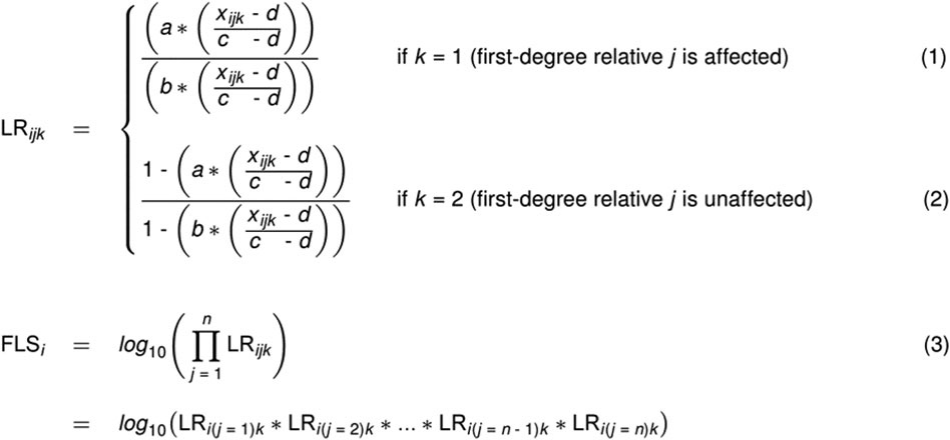
FLS algorithm designed by Verdoux et al. (1996) consisting of three formulas: (1) a formula determining a LR for whether a participant *i* is at familial risk for depression and/or anxiety or not, given that a first-degree relative *j* of age *x_ijk_* is affected (*k* = 1); (2) a formula determining a LR for whether a participant *i* is at familial risk for depression and/or anxiety or not, given that a first-degree relative *j* of age *x_ijk_* is unaffected (*k* = 2); and (3) a formula calculating the FLS for a participant *i* by multiplying all LR of their affected (*k* = 1) and unaffected (*k* = 2) first-degree relatives into one overall LR and taking common logarithm of this overall LR, with *j* the indicator for a first-degree relative and *n* the total number of first-degree relatives for a participant *i*. In these LR, *a* reflects the lifetime prevalence of depression and/or anxiety for persons with FH+ (i.e. 0.50; Micco et al. 2009; Rasic et al. 2014), *b* reflects the lifetime prevalence of depression and/or anxiety for persons with FH− (i.e. 0.134; De Graaf et al. 2012; Verdoux et al. 1996), and *c* and *d* reflect the respectively upper and lower limit of the disorders' age range in which most first onsets appear (65 and 5 respectively; De Graaf et al., 2012).

In the calculation of the FLS, likelihood ratios (LR) were determined for whether a participant *i* is at familial risk for depression and/or anxiety or not, given that a first-degree relative *j* of age *x_ijk_* is *affected* (*k* = 1; see Fig. 1, Formula 1). Similarly, LR were determined for whether a participant *i* is at familial risk for these conditions or not, given that a first-degree relative *j* of age *x_ijk_* is *unaffected* (*k* = 2; see Fig. 1, Formula 2). In these LR, *a* reflects the lifetime prevalence of depression and/or anxiety for persons with FH+ of these conditions (i.e. 0.50; Micco et al. 2009; Rasic et al. 2014), *b* reflects the lifetime prevalence of depression and/or anxiety for persons with FH− of these conditions (i.e. 0.134; De Graaf et al. 2012; Verdoux et al. 1996), *x_ijk_* reflects the age of a first-degree relative *j*, and *c* and *d* reflect the respectively upper and lower limits of the disorders' age range in which most first onsets appear (65 and 5, respectively; De Graaf et al., 2012). In the absence of a better estimate for parameter *b*, which is not precisely known in the literature, we assumed that this estimate was half of the lifetime prevalence of the disorders in the general population in the Netherlands (i.e. 26.8%; De Graaf et al., 2012). This is in line with what was done by the researchers that developed the FLS algorithm (Verdoux et al., 1996) and by other researchers that used the algorithm in later studies (Hillegers et al., 2004; Wals et al., 2004). A LR was calculated for each first-degree relative. Then, for each participant, individual LR of all their first-degree relatives were multiplied to yield an overall LR for the extent to which that participant is at familial risk for depression and/or anxiety or not. As the overall LR is likely to be highly skewed, the FLS is defined as the common logarithm of this overall LR (with *j* the indicator for a first-degree relative and *n* the total number of first-degree relatives for a participant *i*; see Fig. 1, Formula 3).

#### Genetic vulnerability – polygenic risk score (PRS)

A PRS for major depression was built using genotype data in NESDA, for which details on measurement and quality control have been previously reported (Mbarek et al., 2017). The PRS was built leveraging summary statistics from the large genome-wide association study (GWAS) of major depression from the Psychiatric Genomics Consortium (PGC; Wray et al., 2018), including 135 458 cases and 344 901 controls. Details of SNP selection and PRS building can be found extensively described elsewhere (Milaneschi et al., 2019). The PRS including ~1.M genetic variants was built according to LDpred method (Vilhjálmsson et al., 2015) and was standardized to aid interpretation of the results.

#### Clinical vulnerabilities

Past week severity of symptoms was measured with the Inventory of Depressive Symptomatology-Self Report (IDS-SR; Rush, Gullion, Basco, Jarrett, & Trivedi, 1996) for depression and via the Beck Anxiety Inventory (BAI; Beck, Epstein, Brown, & Steer, 1988) for anxiety. For each available wave, IDS-SR and BAI sum-scores were computed with higher scores indicating an increased number and severity of symptoms. As depression/anxiety symptoms have been shown to have relatively high and similarly high 9-year temporal stability in NESDA [range intraclass correlations (ICC) 0.54–0.73; range consistency 0.64–0.74; Struijs et al., 2020], sum-scores were averaged over all available previous waves at which the variables were assessed in order to best reflect participants' overall condition rather than their current state (see Table 1 for dependent variable characteristics). Disease burden of depression and/or anxiety was measured using the life-chart method, which is a calendar-based standardized interview that assessed the presence and severity of symptoms over a period of time before moment of administration (Lyketsos, Nestadt, Cwi, Heithoff, & Eaton, 1994). The time frame for the Life-chart was ‘in the past five years’ at baseline and ‘since the last assessment’ at follow-up assessments. Disease burden was expressed as the percentage of time spent with depression/anxiety symptoms over 14 years. To determine the earliest age of onset of depression/anxiety, CIDI data (Wittchen, 1994) from all available face-to-face assessments were used.

**Table 1.** Measurement characteristics for clinical and psychosocial vulnerabilities

	Variable		
Vulnerabilities	Type	Range	Time points of assessment
Severity of depression symptoms (IDS-SR)	Sum-score (28 items)^a^	0–84	Baseline, 1-, 2-, 4-, 6-, 9-year follow-up
Severity of anxiety symptoms (BAI)	Sum-score (21 items)^a^	0–63	Baseline, 1-, 2-, 4-, 6-, 9-year follow-up
Disease burden (Life-chart)	% time spent with depression/anxiety symptoms in past 14 years	0–100	Baseline, 2-, 4-, 6-, 9-year follow-up
Age of onset of depression/anxiety (CIDI, version 2.1)	Earliest age of onset (in years)	N/A	Baseline, 2-, 4-, 6-, 9-year follow-up
Neuroticism (NEO-FFI)	Subdomain sum-score (12 items)^a^	12–60	Baseline, 2-, 4-year follow-up
Introversion (NEO-FFI)	Subdomain sum-score (12 items)^a^	12–60	Baseline, 2-, 4-year follow-up
External locus of control (Mastery Scale)	Sum-score (5 items)^a^	5–25	Baseline, 2-, 4-, 6-, 9-year follow-up
Hopelessness (LEIDS-R)	Subscale sum-score (5 items)^a^	4–20	Baseline, 2-, 4-, 6-, 9-year follow-up
Rumination (LEIDS-R)	Subscale sum-score (6 items)^a^	4–24	Baseline, 2-, 4-, 6-, 9-year follow-up
Anxiety sensitivity (ASI)	Sum-score (16 items)^a^	0–64	Baseline, 2-, 9-year follow-up
Childhood trauma (CTI)	Sum-score of number and frequency of events	0–8	Baseline
Recent^b^ negative life events (LTE)	Total number of past 3-year events (12 items)	0–12	9-year follow-up

IDS-SR, Inventory of Depressive Symptomatology-Self Report; BAI, Beck Anxiety Inventory; CIDI, Composite International Diagnostic Interview; NEO-FFI, NEO Five Factor Inventory; LEIDS-R, Leiden Index of Depression Sensitivity-Revised; ASI, Anxiety Sensitivity Index; CTI, Childhood Trauma Interview; LTE, List of Threatening Experiences.

a (Subscale/subdomain) sum-scores were averaged over all available NESDA waves.

b Past 3-year.

#### Psychosocial vulnerabilities

As was done for severity of symptoms and in line with the previously found relatively high and similarly high 9-year temporal stability of personality traits and depressive/anxiety cognitions in NESDA (range ICCs 0.53–0.80; range consistency 0.60–0.75; Struijs et al., 2020), where repeated measures at previous assessment waves were available, sum-scores scores were averaged to best represent participants' overall condition.

The Dutch NEO-Five Factor Inventory (NEO-FFI; Hoekstra, Ormel, & Fruyt, 1996) was used to assess two personality domains: neuroticism and introversion. The Mastery Scale (Pearlin & Schooler, 1978) was used to assess external locus of control. Hopelessness and rumination were measured using two subscales of the revised Leiden Index of Depression Sensitivity (LEIDS-R questionnaire; Van Der Does, 2002), which assesses cognitive reactivity to sadness. The Anxiety Sensitivity Index (ASI; Peterson & Reiss, 1992) was used to measure anxiety sensitivity, which represents the extent to which persons fear potentially negative consequences of anxiety related symptoms and sensations. Childhood trauma before the age of 16 (i.e. emotional neglect, psychological abuse, physical abuse, and sexual abuse) was assessed using the Childhood Trauma Interview (CTI; De Graaf, Bijl, Smit, Vollebergh, & Spijker, 2002). A sum-score was computed from the experienced number and frequency of childhood trauma events. The List of Threatening Experiences (LTE; Brugha, Bebbington, Tennant, & Hurry, 1985) was used to assess the total number of recent (i.e. past 3-year) exposures to serious negative life events (e.g. death of a loved one or loss of a job).

### Statistical analyses

Point-biserial correlations were calculated between FH and the FLS, and between FH and the PRS. A Pearson correlation was calculated between the FLS and the PRS. Analyses including the PRS were based on a smaller sample (*N* = 1217, due to missing genetic data and/or non-European ancestry; Reisberg, Iljasenko, Läll, Fischer, & Vilo, 2017). Then, the extent to which the FLS and FH were associated with each of the dependent variables (clinical/psychosocial vulnerabilities) was tested by performing two linear regression models for each dependent variable: one with FLS and one with FH as the independent variable and adjusted for age, gender, and years of education. Next, we investigated whether the FLS was associated with each of the dependent variables over and above FH. As a first step, (unstandardized) FLS residuals were saved from a linear regression analysis regressing FLS on FH. As a second step, we examined whether this residualized FLS was associated with each of the clinical and psychosocial vulnerabilities using linear regression analyses adjusted for age, gender, and years of education of the participant.

The Benjamini–Hochberg procedure with a false discovery rate of 5% was used to correct for multiple testing (Benjamini & Hochberg, 1995). Raw *p* values were reported. All analyses were performed in SPSS, version 24 (IBM Corp., Armonk, NY, USA). The code for the FLS calculation and the analyses of this paper are published online on the Open Science Framework (https://osf.io/gbj3z/files/).

## Results

**Fig. 2.**
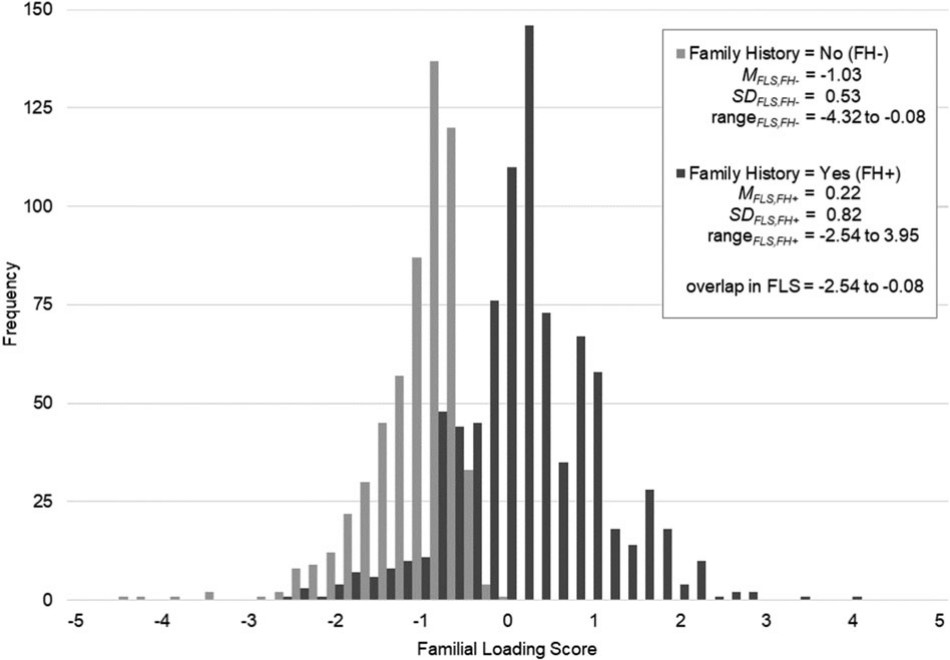
Frequency distribution of the FLS for depression and/or anxiety in lifetime-affected persons (*N* = 1425), segmented across FH Yes (FH+)/No (FH−) groups. A higher FLS reflects a higher familial load. FH, family history; FLS, familial loading score; *M*, mean; s.d., standard deviation.

Of the 1425 lifetime-affected persons included (66.6% female, 26–75 years), 59.8% reported FH+ of depression and/or anxiety (see Table 2 for sample characteristics). Persons with FH+ were younger (*p* < 0.001) and were from larger families (*p* < 0.001) than persons with FH−, but did not differ in gender (*p* = 0.057) and years of education (*p* = 0.484). As expected, a strong, significant correlation was found between the FLS and FH (*r* = 0.65, *p* < 0.001) and persons with FH+ had a higher FLS than persons with FH−, *t*(1423) = −32.16, *p* < 0.001. FLS was approximately normally distributed and even within FH Yes/No groups a substantial amount of variability in scores was observed (Fig. 2). Moreover, persons with FH+ and persons with FH− showed a considerable overlap in FLS.

**Table 2.** Socio-demographics, clinical and psychosocial vulnerabilities, and family characteristics of participants (*N* = 1425)

Sample characteristics	*N*/*M*	%/s.d.
Socio demographics		
Female (*N*; %)	949	66.60
Age (*M* years; s.d.)	51.12	12.82
Years of education (*M*; s.d.)	12.84	3.28
Clinical vulnerabilities		
Severity of depression symptoms (*M*; s.d.)	18.65	10.30
Severity of anxiety symptoms (*M*; s.d.)	10.15	7.36
Disease burden (*M* % of time with depression/anxiety symptoms in past 14 years; s.d.)	46.77	32.77
Age of onset of depression/anxiety (*M* years; s.d.)	21.55	13.74
Psychosocial vulnerabilities		
Neuroticism (*M*; s.d.)	35.83	7.30
Introversion (*M*; s.d.)	35.24	6.50
External locus of control (*M*; s.d.)	12.28	3.71
Hopelessness (*M*; s.d.)	4.31	3.44
Rumination (*M*; s.d.)	8.42	4.00
Anxiety sensitivity (*M*; s.d.)	28.60	7.65
Childhood trauma (*M*; s.d.)	1.68	2.09
Recent^a^ negative life events (*M* no.; s.d.)	1.86	1.44
Family characteristics		
First-degree relatives per family (*M* no.; s.d.)	4.51	2.08
First-degree relatives per family with lifetime depression and/or anxiety (*M* no.; s.d.)	0.97	1.08
Age sibling(s)^b^ (*M* years; s.d.)	50.24	13.60
Age mother (*M* years; s.d.)	73.06	13.00
Age father (*M* years; s.d.)	71.38	11.89
FH of depression and/or anxiety (*N*; % yes)	852	59.79
FLS for depression and/or anxiety (*M*; s.d.)	−0.28	0.94

s.d., standard deviation; *M*, mean; depression, major depressive disorder or dysthymia; anxiety, panic disorder with or without agoraphobia, generalized anxiety disorder, social phobia, agoraphobia.

*Note*. Sample sizes vary slightly due to marginally missing data.

a Past 3-year.

b Valid data on *N* = 1311 as 114 participants indicated that they do not have any siblings.

### Associations of familial risk with genetic, clinical, and psychosocial vulnerabilities

Weak positive correlations were found between the PRS and FLS (*r* = 0.07, *p* = 0.023) and between the PRS and FH (*r* = 0.05, *p* = 0.081). With respect to associated clinical vulnerabilities, results from linear regression analyses showed that lifetime-affected persons with higher FLS for depression and/or anxiety had more severe symptoms of depression (*β* = 0.07, *p* = 0.010) and anxiety (*β* = 0.07, *p* = 0.016), higher disease burden (*β* = 0.10, *p* = 0.001), and younger age of onset (*β* = −0.09, *p* = 0.001; Table 3). FLS for depression and/or anxiety was also associated with several psychosocial vulnerabilities: those with higher FLS showed higher levels of neuroticism (*β* = 0.07, *p* = 0.021), rumination (*β* = 0.12, *p* < 0.001), and experienced more childhood trauma (*β* = 0.13, *p* < 0.001). We found some of these associations with clinical and psychosocial vulnerabilities for the dichotomous FH indicator, but associations were not significant for severity of symptoms of depression (*β* = 0.02, *p* = 0.521) and anxiety (*β* = 0.02, *p* = 0.350), and neuroticism (*β* = 0.05, *p* = 0.073). Further analyses using separately the more simple continuous indicators included in the FLS, such as number and proportion of affected first-degree relatives, showed a lower number of significant associations as compared to the use of the composite FLS index: associations with severity of symptoms were not significant with both single indicators, and number of affected relatives was additionally not associated with the PRS and neuroticism (results not shown).

**Table 3.** Adjusted^a^ associations of FLS and FH Yes/No indicators for depression and/or anxiety with clinical and psychosocial vulnerabilities (*N* = 1425)

	Measures of familial risk for depression and/or anxiety					
Vulnerabilities	FLS			FH (Yes/No)		
	*b*	[95% CI*_b_*]	*β*	*b*	(95% CI*_b_*)	*β*
Clinical						
Severity of depressive symptoms	0.80	[0.19 to 1.42]	0.07*	0.35	[−0.72 to 1.43]	0.02
Severity of anxiety symptoms	0.54	[0.10 to 0.98]	0.07*	0.37	[−0.40 to 1.13]	0.02
Disease burden^b^	3.30	[1.30 to 5.30]	0.10**	4.96	[1.47 to 8.45]	0.07**
Age of onset of depression/anxiety	−1.31	[−2.09 to −0.52]	−0.09**	−2.56	[−3.93 to −1.19]	−0.09***
Psychosocial						
Neuroticism	0.52	[0.08 to 0.97]	0.07*	0.71	[−0.07 to 1.48]	0.05
Introversion	0.24	[−0.15 to 0.63]	0.03	0.26	[−0.42 to 0.94]	0.02
External locus of control	0.11	[−0.11 to 0.34]	0.03	0.28	[−0.11 to 0.67]	0.04
Hopelessness	0.19	[−0.02 to 0.40]	0.05	0.31	[−0.05 to 0.68]	0.04
Rumination	0.49	[0.25 to 0.73]	0.12***	0.74	[0.32 to 1.17]	0.09***
Anxiety sensitivity	0.26	[−0.21 to 0.73]	0.03	0.29	[−0.53 to 1.10]	0.02
Childhood trauma	0.29	[0.17 to 0.42]	0.13***	0.40	[0.18 to 0.62]	0.09***
Recent^c^ negative life events	0.06	[−0.03 to 0.15]	0.04	0.16	[0.003 to 0.31]	0.05

*b*, unstandardized regression coefficient; 95% CI*_b_*, 95% confidence interval of *b*; *β*, standardized regression coefficient.

*Note.* Sample sizes vary slightly due to marginally missing data. Significance is indicated with raw *p* values using the Benjamini–Hochberg procedure (Benjamini & Hochberg, 1995) with a false discovery rate of 5% to correct for multiple testing.

a All linear regression models were adjusted for age, gender, and years of education.

b Measured as mean % of time with depression/anxiety symptoms in past 14 years.

c Past 3-year.

***p < 0.001; **p < 0.01; *p < 0.05.

After regressing out the effect of FH from the FLS (*β* = 0.65, *p* < 0.001), the residualized FLS showed still significant associations with severity of symptoms of depression (*β* = 0.08, *p* = 0.005) and anxiety (*β* = 0.07, *p* = 0.021), rumination (*β* = 0.06, *p* = 0.002), and childhood trauma (*β* = 0.08, *p* = 0.008). Thus, the FLS was associated with severity of symptoms, rumination, and childhood trauma, over and above FH. No significant associations were found between the residualized FLS and other clinical and psychosocial vulnerabilities. As could be expected from the small significant correlation between the FLS and PRS, regressing out the effect of the PRS from the FLS (*β* = 0.07, *p* = 0.023) did not change results: the residualized FLS showed still significant associations with all vulnerabilities that previously showed significant associations with the FLS when the PRS was not regressed out (see online Supplementary Appendix S4).

## Discussion

The present study showed that a continuous measure of familial risk (FLS), incorporating family- and disorder-specific characteristics (e.g. family size and prevalence), was associated with higher genetic vulnerability for major depression and several clinical/psychosocial vulnerabilities for depression and anxiety. Lifetime-affected persons with a higher depression and/or anxiety FLS had more severe symptoms, higher disease burden, and earlier age of onset, as well as higher levels of neuroticism, rumination, and childhood trauma, indicating an overall more unfavorable disease profile. Importantly, the continuous FLS was associated with more severe symptoms, rumination, and childhood trauma over and above the dichotomous FH measure. Overall, our results suggest that FLS is a more comprehensive indicator of familial risk by detecting genetic, clinical and psychosocial vulnerabilities for depression and anxiety that are (partly) unidentified by the dichotomous measure.

### Associations of familial risk with genetic, clinical, and psychosocial vulnerabilities

Lifetime-affected persons with high familial risk had higher scores on an established index for major depression liability in genetics (PRS), which was consistent with previous findings indicating that familial risk (partly) represents an underlying genetic vulnerability for depression and anxiety (Smoller, 2016).

Furthermore, supporting most previous studies, a higher FLS was associated with younger age of onset (Hillegers et al., 2004; Husain et al., 2008; Seguí et al., 1999; Tozzi et al., 2008; Wals et al., 2004) as well as more severe symptoms and higher disease burden of depression and anxiety (Holma et al., 2011; Klein et al., 2008). Whereas psychopathology in lifetime-affected persons with low familial risk for depression and/or anxiety may be mainly explained by external factors (e.g. by negative life events), persons with high familial risk are likely exposed to additional risk factors (besides the inherited genetic risk) that are associated with growing up with an affected sibling or parent (Lukens & Thorning, 2011). For instance, parental neglect (e.g. as a result of increased needs of an affected sibling; Del Rosario & Keefe, 2003) may further increase a person's vulnerability for poor outcome.

Additionally, our findings show that the impact of familial risk extends to a wide range of psychosocial vulnerabilities. In addition to neuroticism, which was previously found to be associated with familial risk in one study (Holma et al., 2011) but not in another (Duggan et al., 1998), we revealed two additional associated psychosocial vulnerabilities – rumination and childhood trauma. One explanation for this finding is that familial risk may indirectly trigger depression and anxiety via neuroticism, rumination (Du Pont, Rhee, Corley, Hewitt, & Friedman, 2019), and childhood trauma (Brietzke et al., 2012; Jansen et al., 2016). For instance, with regard to childhood trauma, parental psychopathology may have a negative impact on offspring functioning via an increased risk for adverse (i.e. more hostile, negative, and disengaged/withdrawn) parenting behavior (National Research Council & Institute of Medicine, 2009). Research has shown that parents account for 80% of the identified perpetrators of childhood trauma (i.e. emotional/physical maltreatment; Hovens et al., 2010). With respect to rumination, an overcontrolling parenting style due to parental psychopathology may increase the risk for future rumination in offspring (Hilt, Armstrong, & Essex, 2012; Spasojevíc & Alloy, 2002).

### How to construct familial risk for depression and anxiety – FLS *versus* FH

Our findings showed a substantial amount of variability in FLS, even within FH groups, and a considerable overlap in FLS between FH groups. Overall, this indicates that familial risk is a complex and dimensional construct, and that a simple dichotomization may not fully capture the heterogeneity in familial risk. Crucially, this indicates that if lifetime-affected persons have one or more family members with depression and/or anxiety (FH+), information about the number of affected and unaffected first-degree relatives they have, the age of these relatives, in what age range most first onsets appear, and what the lifetime prevalence of the disorders is, additionally contributes to the degree of familial risk. Similarly, lifetime-affected persons with FH− still showed substantial variability in FLS even though their first-degree relatives were all reported to be unaffected.

In further support of our hypothesis that a continuous FLS provides a more comprehensive indicator of familial risk than a dichotomous FH measure, the FLS was associated with severity of depression/anxiety symptoms, rumination, and childhood trauma when the effect of FH was regressed out of FLS. Additionally, the FLS was able to pick up on a genome-wide PRS for major depression, severity of depression/anxiety symptoms, and neuroticism, whereas FH was not. While there are currently no studies available regarding the various psychometric properties of the FLS, for instance reliability, previous studies have indicated predictive validity of the FLS for several clinical outcomes (e.g. more severe symptoms or earlier age of onset) in psychotic patients (Verdoux et al., 1996) and children of bipolar parents (Hillegers et al., 2004; Wals et al., 2004). Our results provide further evidence of predictive validity of the FLS for several genetic, clinical, and psychosocial vulnerabilities, even after information on FH has been accounted for.

Our findings may be explained by the fact that by default the dichotomous FH indicator has less statistical power to differentiate in terms of such vulnerabilities as compared to the continuous FLS (in particular for associations with the PRS, effect sizes were rather similar; Cohen, 1983). However, a previous community sample study found no difference in predictive validity (of disorder status) between a dichotomous FH and several continuous scores (e.g. the number and proportion of affected relatives; Milne et al., 2008). Moreover, sensitivity analyses suggest that more simple continuous indicators included in the FLS, such as number and proportion of affected first-degree relatives, were also outperformed by the FLS. Specifically, associations with severity of symptoms were not significant for both single indicators, and number of affected relatives was additionally not associated with the PRS and neuroticism. Another explanation is that besides having more statistical power, a continuous FLS may be able to capture more information by taking into account several family- and disorder-specific characteristics and is therefore likely to better reveal a person's vulnerability for psychopathology (Derks et al., 2009). Together, both explanations may explain why, in contrast to findings of the present study, several previous studies failed to find associations between familial risk (measured as FH) and liability for major depression (as indicated by a PRS; Van Loo et al. 2018; Verduijn et al. 2017), number of depressive episodes, age of onset (Johnson et al., 2000), severity of depression symptoms (Lamers et al., 2011a), neuroticism (Duggan et al., 1998), and stressful life events (Manfro et al., 1996).

### Strengths and limitations

Strengths of the present study include the relatively large community-based sample recruited from diverse settings; the extensive FH assessment (including several validating questions) and diagnostic interviews including the full spectrum of depressive and/or anxiety disorders; the wide variety of assessed genetic, clinical, and psychosocial vulnerabilities; the adequate correction for multiple testing; and the use of a PRS based on a large international consortium that was built using the new LDpred method, which has shown an improved predictive performance compared with other methods (Vilhjálmsson et al., 2015).

Some limitations should be noted as well while interpreting the results. First, the present study performed cross-sectional analyses as only earlier assessment waves were available for clinical and psychosocial vulnerabilities. Thus, no conclusions can be drawn on the direction of associations between familial risk and genetic, clinical, and psychosocial vulnerabilities. Prospective longitudinal studies need to confirm the suggested underlying mechanisms of familial transmission. Overall, effect sizes for FLS were rather small underscoring the need to establish the clinical relevance of this familial risk measure. Recent studies have indicated that psychiatric disorders are transmitted from one generation to the next with little specificity (Dean et al., 2018; Martel et al., 2017; McLaughlin et al., 2012). In this study, FLS/FH indicators were restricted to familial risk for depressive and anxiety disorders as FH information on other psychiatric disorders was not assessed systematically in NESDA. Future studies should look at a broad range of psychiatric disorders in relatives in order to determine cross-disorder transmissions related to the FLS. In addition, FH information was acquired indirectly by interviewing participants on their first-degree relatives. Nevertheless, using indirect interviews rather than examining relatives in person is less expensive and time consuming, making it a convenient method of FH assessment both in research and clinical practice (Hardt & Franke, 2007). Although the validating information on receiving treatment or medical attention will have likely prevented overestimation of familial risk as found in an earlier NESDA study (Lamers et al., 2011a), we did not have information on affected relatives that never sought treatment, which limits generalizability to relatives with milder problems. Moreover, there was no data on second-degree relatives (such as grandparents), which may have resulted in further imprecision. However, the effect of familial risk due to second-degree relatives has been shown to be substantially smaller than the risk due to first-degree relatives (e.g. Isomura et al. 2015; Weissman et al. 2016). The overlap between facets of neuroticism and depression/anxiety symptoms (Luciano et al., 2018; Ormel, Rosmalen, & Farmer, 2004) may have resulted in a slight overestimation of the magnitude of the association between neuroticism and the FLS for depression and/or anxiety. However, previous evidence indicates that neuroticism and depression/anxiety are not completely overlapping measures of the same underlying liability but are (at least partly) different constructs, as shown in recent genomic (Adams et al., 2019) and self-report studies (Uliaszek et al., 2009). As adequate PRS for anxiety disorders are still lacking (Walter et al., 2013), we were limited to comparing associations between FLS/FH and a PRS for major depression only. However, considering the high comorbidity (Lamers et al., 2011b) and shared etiology of depression and anxiety (Mathew et al., 2011), it is likely that results would have been similar when PRS for anxiety disorders were used.

## Conclusions and implications

In research and clinical practice, information about familial risk for depression and anxiety is often assessed using a single question about the presence of FH and constructed as a simple, dichotomous indicator. As such, this measure has likely led to an overestimation of familial risk, potentially diluting associations with clinical outcomes in previous studies, and may not fully capture the liability to depression and anxiety. Although associations with genetic, clinical, and psychosocial vulnerabilities were small, our findings contribute to the literature on familial risk of common mental health disorders: In lifetime-affected persons, the impact of a continuous FLS seems to extend to a wide range of different dimensions of familial transmission of depression and anxiety. Moreover, when indicators of familial risk are based on extensive FH interviews, our results suggest that although FH is an informative indicator of familial risk in terms of associated vulnerabilities, a continuous FLS is even more so and may better capture the heterogeneity of familial risk in lifetime-affected persons. This potentially underscores the importance of using a continuous FLS rather than a dichotomous FH indicator of familial risk for depression and anxiety. Thus in research, FH information should be asked out extensively and an online tool could be developed that automatically implements the FLS algorithm so that it can be easily applied in practice (e.g. embedded in a website, by only requiring the number of first-degree relatives, their age, and whether or not they are affected to be entered; see code on the Open Science Framework).

## Supplementary material

For supplementary material accompanying this paper visit https://doi.org/10.1017/S0033291720002299. click here to view supplementary material
