# Supplementary material for: Familial risk for depressive and anxiety disorders: associations with genetic, clinical, and psychosocial vulnerabilities
Source: Psychol Med. 2020 Jul 6;52(4):696–706. doi: 10.1017/S0033291720002299 (PMC8961330; doi:10.1017/S0033291720002299)
Supplement: Supplementary file 1 [file S0033291720002299sup001.docx]

# Supplementary Material

# Appendix S1. Amount of missing data per wave

**Table S1.** Number of participants by number of waves participated out of a total of six waves (baseline, 1-, 2-, 4-, 6-, and 9-year follow-up)

| # waves participated | *N* | % |
| --- | --- | --- |
| 1 | 0 | 0.0 |
| 2 | 2 | 0.1 |
| 3 | 9 | 0.6 |
| 4 | 36 | 2.5 |
| 5 | 187 | 13.1 |
| 6 | 1191 | 83.6 |
| **Total** | 1425 | 100.0 |

# Appendix S2. Exact questions from Family Tree Inventory (FTI)

## Broad, simple questions

### Depression

1. Did your (mother, father, brother(s) and/or sister(s)) ever have a depressive episode?

- Yes
- No
- Don’t know

### Anxiety

1. Did your (mother, father, brother(s) and/or sister(s)) ever complain about sudden anxiety or panic attacks?

- Yes
- No
- Don’t know

## Validating follow-up questions

### Depression

1. Did your (mother, father, brother(s) and/or sister(s)) ever have a period in which it appeared as though he/she was not interested anymore in the things he/she usually cared about, or a period in which he/she could not enjoy things anymore?
   - Yes
   - No
   - Don’t know
2. Was your (mother, father, brother(s) and/or sister(s)) ever treated for a depression by a professional care provider (general practitioner, RIAGG, psychologist, psychiatrist)?
   - Yes, general practitioner
   - Yes, RIAGG, psychologist, psychiatrist
   - No
   - Don’t know
3. Did your (mother, father, brother(s) and/or sister(s)) ever use medication for these complaints?
   - Yes
   - No
   - Don’t know
4. Was your (mother, father, brother(s) and/or sister(s)) ever admitted (to a hospital/psych ward) for these complaints?
   - Yes
   - No
   - Don’t know
5. Did your mother, father, brother(s) and/or sister(s)) ever undergo ECT during this admission (to the hospital/psych ward)?
   - Yes
   - No
   - Don’t know

### Anxiety

1. Did your (mother, father, brother(s) and/or sister(s)) ever suffer from a period of excessive anxiety or concern (including excessive concern about physical health and fear of social situations)?
   - Yes
   - No
   - Don’t know
2. Did the anxiety or concern cause restrictions, for example, that your (mother, father, brother(s) and/or sister(s)) started to avoid situations, or could only withstand situations with great difficulty or with the help of others?
   - Yes
   - No
   - Don’t know
3. Was your (mother, father, brother(s) and/or sister(s)) ever treated for anxiety complains or panic attacks by a professional care provider (general practitioner, RIAGG, psychologist, psychiatrist)?
   - Yes, general practitioner
   - Yes, RIAGG, psychologist, psychiatrist
   - No
   - Don’t know
4. Did your (mother, father, brother(s) and/or sister(s)) ever use medication for anxiety complaints or panic attacks?
   - Yes
   - No
   - Don’t know
5. Was your (mother, father, brother(s) and/or sister(s)) ever admitted (to a hospital/psych ward) for anxiety complaints or panic attacks?
   - Yes
   - No
   - Don’t know

# Appendix S3. Calculation of familial loading score for depression and/or anxiety

Fig. S1 shows the filled-out algorithm designed by Verdoux et al. (1996) for the familial loading score (FLS) for depression and/or anxiety. Table S2 shows the exact numbers chosen for parameters *a*, *b*, *c*, and *d* in the algorithm. In the calculation of the FLS, likelihood ratios (LR) were determined for whether a participant *i* is at familial risk for depression and/or anxiety or not, given that a first-degree relative *j* of age *x_ijk_* is *affected* (*k* = 1; see Fig. S1, Formula 1) or given that a first-degree relative *j* of age *x_ijk_* is *unaffected* (*k* = 2, see Fig. S1, Formula 2). For affected first-degree relatives (Formula 1), this was done by dividing the probability that a first-degree relative *j* of age *x_ijk_* is affected if the corresponding participant *i* is *at familial risk* (FH+) by the probability that a first-degree relative *j* of age *x_ijk_* is *affected* if the corresponding participant *i* is *not at familial risk* (FH-). For *unaffected* first-degree relatives (Formula 2), a similar procedure was followed with the probability that a first-degree relative *j* of age *x_ijk_* is *unaffected* corresponding to 1 minus the probability that a first-degree relative *j* of age *x_ijk_* is *affected*. Of note is that the LR for whether a participant *i* is at familial risk or not, given that a first-degree relative *j* of age *x_ijk_* is *affected* (Formula 1), is a set number. In contrast, the LR for whether a participant *i* is at familial risk or not, given that a first-degree relative *j* of age *x_ijk_* is *unaffected* (Formula 2), is dependent on the age of relative *j*.

Such LR were calculated for all first-degree relatives of a participant *i*. Then, for each participant, individual LR of all first-degree relatives of that participant were multiplied to yield an overall LR for the extent to which that participant is at familial risk for depression and/or anxiety or not. As this overall LR is likely to be highly skewed, the FLS is defined as the common logarithm of this overall LR (with *j* the indicator for a first-degree relative and *n* the total number of first-degree relatives for a participant *i*; see Fig. S1, Formula 3).

In the formulas of the algorithm, *a* reflects the lifetime prevalence of a disorder for persons at familial risk (FH+), *b* reflects the lifetime prevalence of a disorder for persons not at familial risk (FH-), *x* reflects the age of a first-degree relative *j*, and *c* and *d* reflect the respectively upper and lower limit of the disorders’ age range in which most first onsets appear (see Table S2 for the exact numbers chosen for parameters *a*, *b*, *c*, and *d* for depression, anxiety, and depression and/or anxiety).

Based on two literature reviews, we assumed that the lifetime prevalence for those at familial risk of was 40% for depression (Rasic, Hajek, Alda, & Uher, 2014) and 36% for anxiety (see Table S2; Micco et al., 2009). For depression and/or anxiety, considering their high comorbidity (Lamers et al., 2011) and shared etiology (Mathew, Pettit, Lewinsohn, Seeley, & Roberts, 2011), we assumed a lifetime prevalence of 50% for those at familial risk.

As the general population includes both persons with (FH+) and persons without (FH-) first-degree relatives that are affected with depressive and/or anxiety disorders, derived lifetime prevalence estimates would overestimate the occurrence of the disorders in persons *not at familial risk*. So, in absence of a better estimate for lifetime prevalence of these disorders in persons with FH- (i.e., parameter *b*, which is not precisely known in the literature), we assumed that this estimate was 9.35% for depression and 7.4% for anxiety, that is, half of the lifetime prevalence of the disorders in the general population in the Netherlands (respectively 18.7% and 14.8%; De Graaf, Ten Have, Van Gool, & Van Dorsselaer, 2012). This is in line with what was done by the researchers that developed the FLS algorithm (Verdoux et al., 1996) and by other researchers that used the algorithm in later studies (Hillegers et al., 2004; Wals et al., 2004). Thus, for depression and/or anxiety, considering their high comorbidity (Lamers et al., 2011) and shared etiology (Mathew et al., 2011), we assumed a lifetime prevalence of 13.4%% for those not at familial risk (i.e., half of the lifetime prevalence of either one or both of the disorders in the Dutch general population, which is approximately 26.8%; De Graaf et al., 2012).

If we change the assumption for parameter *b* from half to one-fourth of the lifetime prevalence of depressive and/or anxiety disorders in the general population, the correlation between the FLSs calculated under the two different assumptions is >.99. Similarly, relaxing the original assumption to three-fourths of the lifetime prevalence of the disorders in the general population also gives a correlation between scores of >.99. Also, as compared to the FLS used in our paper, analyses with the scores calculated under the two adjusted assumptions produce very similar results and did not change our conclusions. Therefore, these sensitivity analyses show that results are robust even under different assumptions of lifetime risk for persons not at familial risk.

Finally, we assumed that the age-range in which most first onsets appear was 10 to 65 years for depression and 5 to 50 years for anxiety (see Table S2; De Graaf et al., 2012). We therefore assumed that the age range in which most first onsets of depressive and/or anxiety disorder appear was 5 to 65 years. Within this age range, the algorithm assumes that the risk of disorder increases linearly with age, from zero risk at the lower limit of the range to lifetime risk at the upper limit of the range. This assumption is in line with the fact that the cumulative risk of disorder is approximately zero during early childhood and increases with age due to the growing exposure time-frame that is inherent to aging (e.g. see Beesdo et al., 2009; Essau, Lewinsohn, Xin, Ho, & Rohde, 2018).


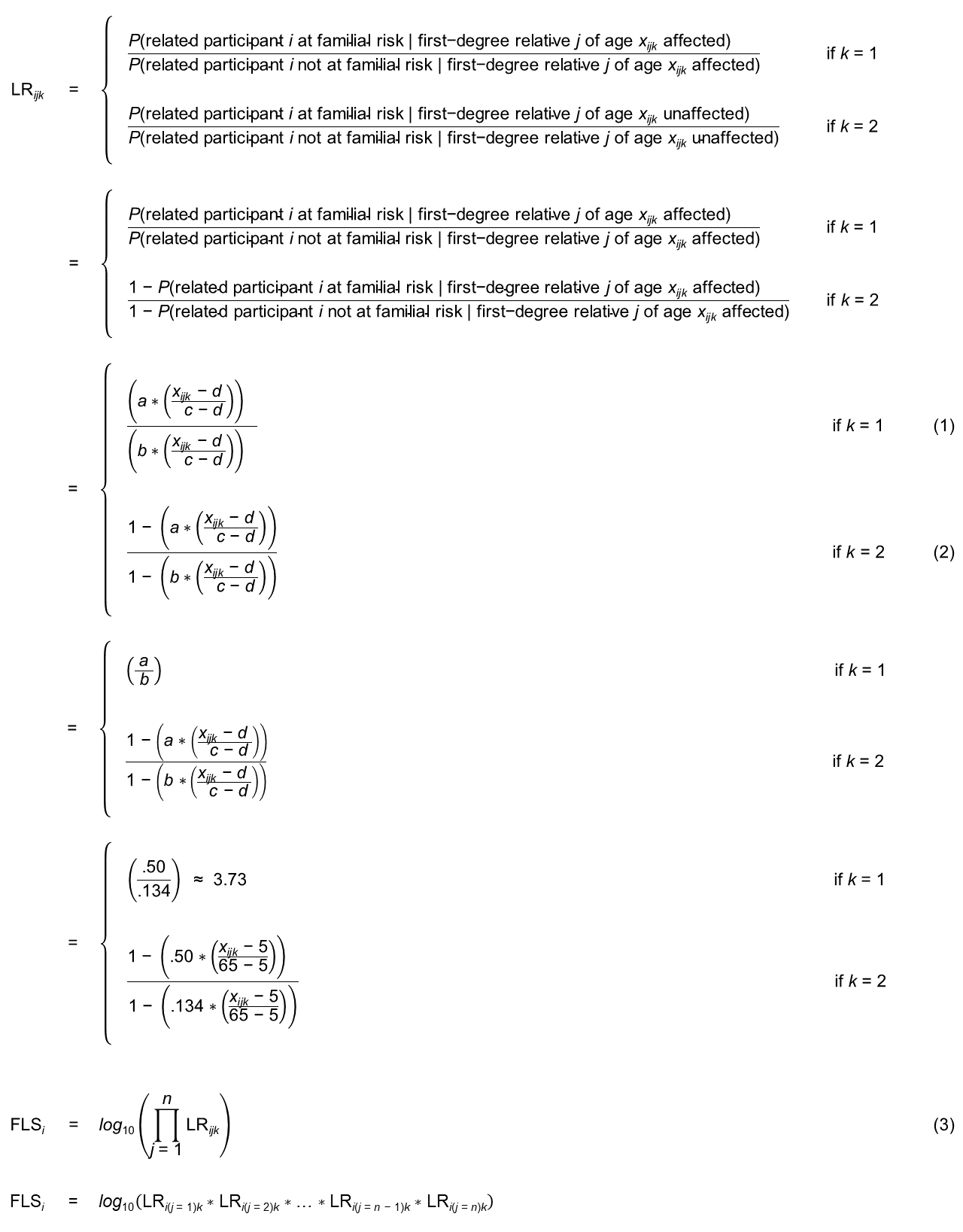


**Fig. S1.** Filled in algorithm designed by Verdoux et al. (1996) using the numbers chosen for parameters *a*, *b*, *c*, and *d* for each disorder (Table S2). The algorithm consists of three formulas: (1) a formula determining a Likelihood Ratio (LR) for whether a participant *i* is at familial risk for depression and/or anxiety or not, given that a first-degree relative *j* of age *x_ijk_* is *affected* (*k* = 1); (2) a formula determining a LR for whether a participant *i* is at familial risk for depression and/or anxiety or not, given that a first-degree relative *j* of age *x_ijk_* is *unaffected* (*k* = 2); and (3) a formula calculating the familial loading score for a participant *i* by multiplying all LR of their affected (*k* = 1) and unaffected (*k* = 2) first-degree relatives into one overall LR and taking common logarithm of this overall LR, with *j* the indicator for a first-degree relative and *n* the total number of first-degree relatives for a participant *i*.

**Table S2.** Numbers chosen for parameters *a*, *b*, *c*, and *d* in the Likelihood Ratio formulas in the FLS algorithm by Verdoux et al. (1996) used to calculate the familial loading score for depression and/or anxiety

|  |  | Lifetime prevalence for those at familial risk | Lifetime prevalence for those not at familial risk | Upper limit of most common age of onset | Lower limit of most common age of onset |
| --- | --- | --- | --- | --- | --- |
| Depression |  | .40 | .0935 | 65 | 10 |
| Anxiety |  | .36 | .074 | 50 | 5 |
|  | Variables in algorithm | *a* | *b* | *c* | *d* |
| Depression and/or anxiety |  | .50 | .134 | 65 | 6 |

### **References**

Beesdo, K., Höfler, M., Leibenluft, E., Lieb, R., Bauer, M., & Pfennig, A. (2009). Mood episodes and mood disorders: Patterns of incidence and conversion in the first three decades of life. *Bipolar Disorders*, *11*(6), 637–649. https://doi.org/10.1111/j.1399-5618.2009.00738.x

De Graaf, R., Ten Have, M., Van Gool, C., & Van Dorsselaer, S. (2012). Prevalence of mental disorders and trends from 1996 to 2009. Results from the Netherlands Mental Health Survey and Incidence Study-2. *Social Psychiatry and Psychiatric Epidemiology*, *47*(2), 203–213. https://doi.org/10.1007/s00127-010-0334-8

Essau, C. A., Lewinsohn, P. M., Xin, J., Ho, M. R., & Rohde, P. (2018). Incidence, recurrence and comorbidity of anxiety disorders in four major developmental stages. *Journal of Affective Disorders*, *228*, 248–253. https://doi.org/10.1016/j.jad.2017.12.014

Hillegers, M. H. J., Burger, H., Wals, M., Reichart, C. G., Verhulst, F. C., Nolen, W. A., & Ormel, J. (2004). Impact of stressful life events, familial loading and their interaction on the onset of mood disorders. *British Journal of Psychiatry*, *185*(2), 97–101. https://doi.org/10.1192/bjp.185.2.97

Lamers, F., Van Oppen, P., Comijs, H. C., Smit, J. H., Spinhoven, P., Van Balkom, A. J. L. M., … Penninx, B. W. J. H. (2011). Comorbidity Patterns of Anxiety and Depressive Disorders in a Large Cohort Study: the Netherlands Study of Depression and Anxiety (NESDA). *Journal of Clinical Psychiatry*, *72*(3), 341–348. https://doi.org/10.4088/JCP.10m06176blu

Mathew, A. R., Pettit, J. W., Lewinsohn, P. M., Seeley, J. R., & Roberts, R. E. (2011). Co-morbidity between major depressive disorder and anxiety disorders: Shared etiology or direct causation? *Psychological Medicine*, *41*(10), 2023–2034. https://doi.org/10.1017/S0033291711000407

Micco, J. A., Henin, A., Mick, E., Kim, S., Hopkins, C. A., Biederman, J., & Hirshfeld-Becker, D. R. (2009). Anxiety and depressive disorders in offspring at high risk for anxiety: A meta-analysis. *Journal of Anxiety Disorders*, *23*(8), 1158–1164. https://doi.org/10.1016/j.janxdis.2009.07.021

Rasic, D., Hajek, T., Alda, M., & Uher, R. (2014). Risk of mental illness in offspring of parents with schizophrenia, bipolar disorder, and major depressive disorder: A meta-analysis of family high-risk studies. *Schizophrenia Bulletin*, *40*(1), 28–38. https://doi.org/10.1093/schbul/sbt114

Verdoux, H., Van Os, J., Sham, P. C., Jones, P. B., Gilvarry, K., & Murray, R. M. (1996). Does familiality predispose to both emergence and persistence of psychosis? A followup study. *British Journal of Psychiatry*, *168*(5), 620–626. https://doi.org/10.1016/0920-9964(96)85389-8

Wals, M., Van Os, J., Reichart, C. G., Hillegers, M. H. J., Ormel, J., Verhulst, F. C., & Nolen, W. A. (2004). Multiple dimensions of familial psychopathology affect risk of mood disorder in children of bipolar parents. *American Journal of Medical Genetics*, *127B*(1), 35–41. https://doi.org/10.1002/ajmg.b.20165

# Appendix S4. Associations with clinical and psychosocial vulnerabilities after regressing out the effect of the polygenic risk score

As could be expected from the small significant correlation between the FLS and the PRS (*r*=.07, *p*=.023), regressing out the effect of the PRS from the FLS (*β*=.07, *p*=.023) did not change the results of associations with clinical and psychosocial vulnerabilities (see Table S3). That is, the residualized FLS showed still significant associations with all outcomes that previously showed significant associations with the FLS when the PRS was not regressed out, namely severity of symptoms of depression (*β*=.06, *p*=.047) and anxiety (*β*=.08, *p*=.016), disease burden (*β*=.09, *p*=.004), age of onset (*β*=-.09, *p*=.002), neuroticism (*β*=.07, *p*=.041), rumination (*β*=.11, *p*<.001), and childhood trauma (*β*=.13, *p*<.001). Thus, the FLS was associated with severity of symptoms, disease burden, age of onset, neuroticism, rumination, and childhood trauma, over and above the PRS.

**Table S3.** Adjusted^a^ associations of the familial loading score with clinical and psychosocial vulnerabilities, after regressing out the effect of the polygenic risk score (*N*=1425)

| Vulnerabilities | Residualized familial loading score after regressing out the polygenic risk score | | |
| --- | --- | --- | --- |
|  | *b* | [95% CI*_b_*] | *β* |
| **Clinical** |  |  |  |
| Severity of depressive symptoms | 0.68 | [0.01, 1.35] | .06* |
| Severity of anxiety symptoms | 0.59 | [0.11, 1.06] | .08* |
| Disease burden^b^ | 3.23 | [1.02, 5.44] | .09** |
| Age of onset of depression/anxiety | -1.33 | [-2.18, -0.48] | -.09** |
| **Psychosocial** |  |  |  |
| Neuroticism | 0.51 | [0.02, 1.00] | .07* |
| Rumination | 0.46 | [0.19, 0.73] | .11*** |
| Childhood trauma | 0.28 | [0.14, 0.42] | .13*** |

*Note.* Sample sizes vary slightly due to marginally missing data. Significance is indicated with raw *p-*values using the Benjamini-Hochberg procedure (Benjamini & Hochberg, 1995) with a false discovery rate of 5% to correct for multiple testing. *b* = unstandardized regression coefficient; 95% CI*_b_* = 95% confidence interval of *b*; *β* = standardized regression coefficient.

^a^ All linear regression models were adjusted for age, gender, and years of education.

^b^ Measured as mean % of time with depression/anxiety symptoms in past 14 years.

*** *p* < .001; ** *p* < .01; * *p* < .05.
